# Role of double-negative autoreactive cells (CD4^−^CD8^−^) in phosphatidylcholine-mediated rheumatoid arthritis: A Mendelian randomization study

**DOI:** 10.1016/j.heliyon.2024.e39946

**Published:** 2024-10-29

**Authors:** Huaguo Zhao, Licheng Ni

**Affiliations:** Department of Orthopedics, Ningbo NO.6 Hospital, 1059 Zhongshan East Road, Ningbo, Zhejiang, 315040, People's Republic of China

**Keywords:** Mendelian randomization, Liposome, Immune cell, Rheumatoid arthritis

## Abstract

**Objective:**

Mendelian randomization (MR) was employed to explore the potential causal relationship between liposomes (LP) and rheumatoid arthritis (RA), with a focus on the mediating roles of immune cells (IC).

**Methods:**

By screening public GWAS data, LP were used as exposure data, RA as outcome data, and IC as mediating factors. The Inverse Variance Weighted (IVW) method was the main analytical technique used in this paper to evaluate causal effects. Additional techniques included the MR-Egger, weighted median, weighted mode, and simple mode methods. Cochran's Q and MR-Egger were utilized for heterogeneity and multi-effect analysis.

**Results:**

Phosphatidylcholine was revealed to enhance the risk of RA by MR analysis (P = 0.013, OR = 1.073, 95%CI = 1.015–1.136). There was no strong evidence that RA could affect liposome changes (IVW: P = 0.705, OR = 0.992, 95%CI = 0.952–1.034). The IVW method showed that increased levels of phosphatidylcholine were notably linked to higher levels of Double-Negative Autoreactive Cells (CD4^−^CD8^−^, DNAC) (P = 0.006, OR = 1.152, 95%CI = 1.041–1.276). The IVW approach showed that increased levels of DNAC were substantially linked to a higher risk of RA (P = 0.001, OR = 1.105, 95%CI = 1.041–1.173). Of the genetically predicted liposomes mediated by DNAC, 19.2 % were found.

**Conclusion:**

The present work established a causal association between LP and RA, and identified a potential mediating influence of IC. However, the specific mechanism of LP triacyl-glycerol and IC on RA is still unclear, and further research is needed.

## 1 Background

A systemic autoimmune illness known as rheumatoid arthritis (RA) is linked to a persistent inflammatory process that can harm extra-articular organs such as the heart, kidneys, lungs, digestive tract, eyes, skin, and nervous system in addition to joints. The disease, of unknown origin, is characterized by inflammation in extra-articular areas. It also involves inflammatory alterations in the synovial tissue, cartilage, and bones of joints [1,2]. There are two types of arthritis commonly discussed in the context of RA: inflammatory arthritis, which is characteristic of RA, and osteoarthritis, which is a type of non-inflammatory arthritis. RA is an autoimmune condition characterized by inflammation, whereas osteoarthritis is a degenerative joint disease resulting from the slow breakdown of joints owing to normal aging and use. An erroneous misconception is commonly made when mistakenly associating RA with diseases caused by crystal deposition, such as gout. Gout is a specific form of arthritis that is not caused by the autoimmune or degenerative processes seen in RA. It occurs due to the build up of urate crystals. RA affects people worldwide, regardless of age, gender, nationality, race, or ethnicity. However, the incidence and prevalence of RA fluctuate over time and are influenced by demographic factors [3]. Regional differences exist, with lower rates observed in countries like Serbia [4], China [5], and France [6], and higher rates in Japan [7] and Argentina [8]. Furthermore, several studies have shown that women are three to five times more likely than males to develop RA.

Liposomes are a versatile delivery mechanism that has been investigated as carriers for encapsulating pharmaceuticals for the treatment of osteoporosis, bone regeneration, gene delivery, and targeted delivery to specific parts of the bone [9]. Liposomal carriers have the potential to increase the effectiveness of medications by enhancing their capacity to be absorbed by the body and their ability to specifically target certain areas. Consequently, this aids in diminishing the frequency of systemic side effects [10]. Enhancing the bioavailability of medications can be accomplished by facilitating absorption, shielding medicines from fast metabolism, and extending their half-lives [11]. Liposomes typically consist of bilayered phospholipids, with phosphatidylcholine being a crucial phospholipid [12].

Current research on liposomes and RA primarily focuses on the use of liposomes as a drug delivery system for treating the disease, but the specific mechanisms of action are still under study [13]. Macrophages and monocytes are important in the pathophysiology of RA, accumulating in the synovial tissue and potentially integrating into the synovium with diverse phenotypes. Additionally, neutrophils play a crucial role, especially where immune complexes activate the complement system, leading to significant tissue damage through the release of their enzymes [14]. However, the relationships between liposomes, immune cells, and RA are yet to be clearly defined in the literature.

Using genetic variation as an instrumental variable (IV), Mendelian randomization (MR) is a theoretical framework used in research to determine the likely causal relationship between exposure and outcomes [15]. The MR Method possesses significant academic significance due to its ability to imitate the results of randomized controlled trials by randomly assigning genetic variation to offspring during meiosis, without the influence of confounding factors or reverse causation [16]. The main goal of this research was to provide new information and strategies for managing and preventing RA by examining the relationship between liposomes, immune cells (IC), and RA. This was achieved using mediated magnetic resonance imaging (mediated MR) and GWAS data.

## 2 Methods

### 2.1 Study design

In this study, the TSMR (Two samples Mendelian Randomization) approach was utilized to examine the causal relationship between liposomes on RA risk, while the two-step mediated Mendelian randomization method was used to ascertain the extent of immune cells' mediating involvement. Liposomes were considered as exposure factors, immune cells as mediators, and instrumental variables were selected based on their significant associations with single nucleotide polymorphisms (SNPs). RA served as the outcome variable. To ensure the validity of MR Causal inference, three fundamental assumptions of instrumental variables (IVs) needed to be met: (i) Genetic variation and exposure are directly correlated; (ii) when taking genetic variation and other confounders into account, there is no correlation between exposure and result; (iii) apart from exposure, genetic variation has no independent effect on the results [16].

### 2.2 GWAS summary data sources

Exposure liposome data and mediator metabolite data were obtained from a publicly accessible GWAS database with exposure data search numbers ranging from GCST90277238 to GCST90277416 for a total of 179 liposomes [17]. The GWAS data included 7174 unrelated Finnish subjects and a total of 849,501 SNPs. The intermediate metabolite data were retrieved from datasets numbered GCST90001391 to GCST90002121 and contained a total of 731 different immune cell data points [18]. There were no overlapping cohorts in the first GWAS on immunological characteristics, which used data from 3757 European people. Twenty-two million SNPs that were genotyped using high-density arrays were imputed using a reference panel based on Sardinian sequences. Outcome data from the FINNGEN RESEARCH PROJECT included 12555 cases and 240,862 controls. All this data was derived from publicly published GWAS data and provided with ethical approval and informed consent.

### 2.3 Instrumental variable selection and data harmonization

First, a significance threshold of P < 1 × 10^−5^ is applied to select SNPs, while ensuring no linkage disequilibrium (LD) exists (R^2^ < 0.001, kb = 10000). Secondly, to satisfy the assumption of independence and exclusivity, SNPs closely associated with metabolites were queried in the PhenoScanner V2 database. In order to guarantee that the chosen SNPs are not connected to any potential confounding factors between exposure and outcome variables, SNPs linked with confounders and outcome variables were carefully removed [19,20]. Furthermore, SNPs inconsistent with exposure and outcome alleles as well as palindromic SNPs with moderate allelic frequencies are excluded from further analysis after rigorous screening. Finally, the F statistic is used to evaluate the instrumental variable's strength: [(N-K-1)/K]/[R^2^/(1-R^2^)], where K represents genetic variation and N denotes sample size. Weak instrumental factors are unlikely to affect MR results if the F value is larger than 10 [21].

### 2.4 MR analysis

The R4.2.3 program was used to perform the statistical studies (http://www.Rproject.org). The weighted mode, weighted median [22], weighted MR-Egger regression, and simple mode [23] were used to evaluate the causative association between RA and 179 liposomes. The primary MR analysis technique employed in this study was the IVW approach. The most accurate estimate of causal association effects is produced by the IVW technique when all chosen SNPs are genuine instrumental variables (IVs) [24].

### 2.5 Mediation effect analysis

**Fig. 1:**
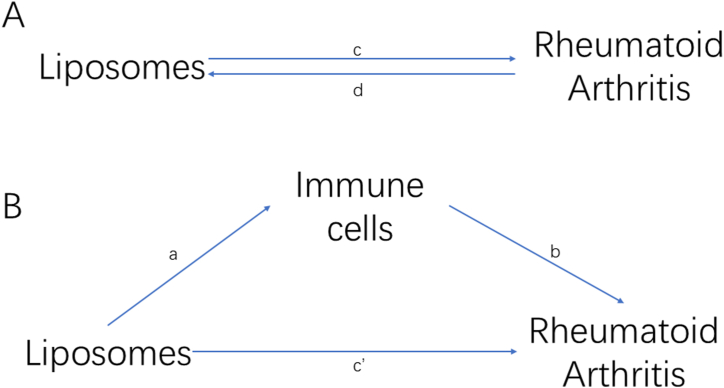
A graph illustrating the correlation of the methods used in this study. (A) The overall effect of Liposomes (LP) and rheumatoid arthritis (RA). c is the total effect of liposome as exposure and RA as outcome; d is the total effect of RA as exposure and liposomes as outcome. (B) The total effects of liposomes and RA are decomposed into: (1) indirect effects, using a two-step method (where A is the effect of liposomes on immune cells, b is the effect of immune cells on RA; The indirect effect is (a × b); (2) Direct influence (c' = c-a × b).

Using a two-step mediated MR analysis, we investigated the possible mediating function of metabolites between liposomes and RA, as well as the magnitude of this mediation (Fig. 1). The total effects of LP on RA (Fig. 1A) can be categorized into direct and indirect effects, each of which is mediated by mediators(Fig. 1B) [25]. The total effect of liposomes on RA was decomposed into i) direct effects of liposomes on RA (c' in Fig. 1B) and ii) indirect effects mediated by liposomes through the mediator (a × b in Fig. 1B). Decomposing the overall effect of liposomes on RA involves evaluating both the direct impact of liposomes on RA and the indirect influence mediated by intermediary metabolites. By dividing the indirect effect by the total effect, we were able to determine the fraction mediated by the mediating influence.

### 2.6 Sensitivity analysis

The Cochran's Q statistic and related P-values were used to assess the heterogeneity of the chosen instrumental variables (IVs). If the null hypothesis is not accepted, the fixed effects IVW technique is substituted by the random effects IVW method. MR-Egger, a recognized approach, is activated when its intercept attains statistical significance to address potential horizontal pleiotropy [[23], [26]]. Furthermore, within the framework of MR-PRESSO technique, we utilized it to identify and eliminate any influential outliers that may have significantly impacted estimation due to horizontal pleiotropy [27]. Scatter plots were employed to evaluate the robustness of study findings against potential anomalous disturbances, complemented by funnel plots to enhance reliability and ensure consistency across studies in observed associations. The results were displayed as odds ratios (ORs) accompanied by the corresponding 95 % confidence intervals (CIs). For every test, a significance level of α = 0.05 was employed.

## 3 Results

### 3.1 Association of liposomes with RA

SNPs with linkage imbalance (R^2^ = 0.001, kb = 10,000) were excluded during the screening of standard SNPs (P < 1 × 10^−5^), and SNPs potentially associated with confounders or outcome variables were excluded through PhenoScanner V2 database (none found). The GWAS data of liposomes showed that 11 kinds of liposomes were correlated with RA (P < 0.05). Phosphatidylcholine (16:1–20:4) had the strongest correlation (P = 0.013). Twenty-one significantly correlated and independent SNPs were identified and extracted for causal analysis.

**Fig. 2:**
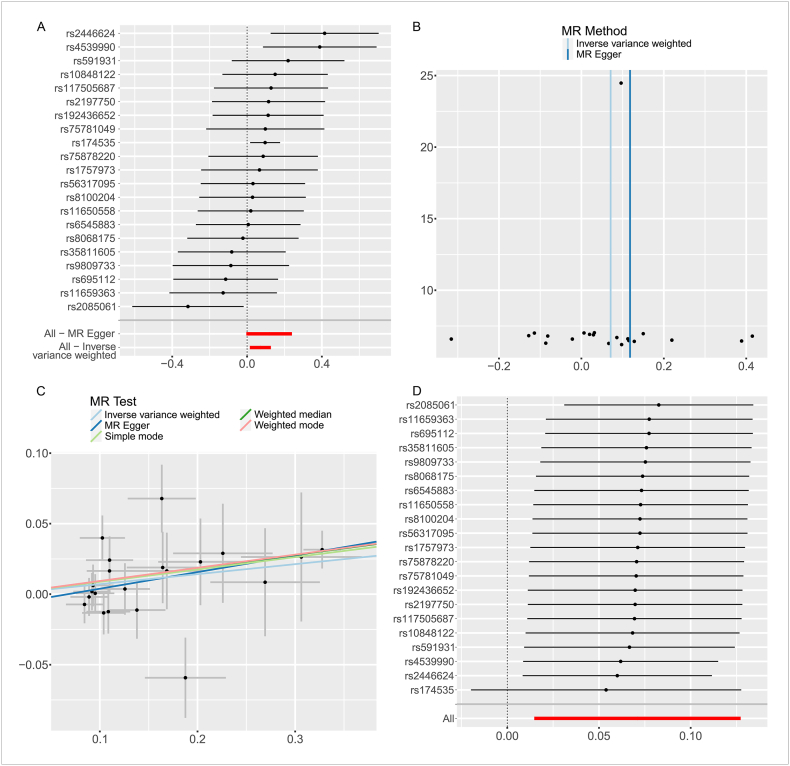
MR Analysis of phosphatidylcholine and RA. A: Leave-one-out forest map; B funnel diagram; C Scatter plot: Lines in black, red, green, and blue represent IVW, MR-Egger, weighted median, and weight mode methods; D Forest map for sensitivity analysis. (For interpretation of the references to colour in this figure legend, the reader is referred to the Web version of this article.)

As shown in Table 1, the IVW approach demonstrated a significant correlation between elevated phosphatidylcholine and an increased risk of developing RA (P = 0.013, OR = 1.073, 95%CI = 1.015–1.136). As a supplement, Weighted mode method as well as Weighted median method also suggested that increases in Phosphatidylcholine were significantly associated with an increased risk of acquiring RA (Weighted median: P = 0.013, OR = 1.097, 95%CI = 1.020–1.181; Weighted mode: P = 0.026, OR = 1.098, 95%CI = 1.017–1.185). In addition, the outcomes of the MR-Egger method and Simple mode method indicate the same causal estimating direction. However, it could not reach statistical significance (MR-Egger:P = 0.073, OR = 1.125, 95%CI = 0.996–1.271; Simple mode: P = 0.240, OR = 1.092, 95%CI = 0.947–1.258)(Fig. 2A–D). Our MR Analysis's findings, however, did not show a reverse causal connection between RA and liposomes. Table 1 displays the IVW method's results (IVW: P = 0.705, OR = 0.992, 95%CI = 0.952–1.034).

**Table 1:** The results of MR analysis.

Exposure	Outcome	MR methods	*SNPs*	*P-Value*	*OR(95%CI)*	*F*
Liposome(Phosphatidylcholine)	Rheumatoid Arthritis	MR Egger	21	0.073	1.125(0.996–1.271)	34.484
		Weighted median	21	0.013	1.097(1.020–1.181)	
		Inverse variance weighted	21	0.013	1.074(1.015–1.136)	
		Simple mode	21	0.240	1.092(0.947–1.258)	
		Weighted mode	21	0.026	1.098(1.017–1.185)	
Rheumatoid Arthritis	Liposome(Phosphatidylcholine)	MR Egger	188	0.095	1.060(0.990–1.135)	37.206
		Weighted median	188	0.970	1.001(0.929–1.080)	
		Inverse variance weighted	188	0.705	0.992(0.952–1.034)	
		Simple mode	188	0.646	0.962(0.816–1.134)	
		Weighted mode	188	0.309	1.036(0.968–1.110)	
Liposome(Phosphatidylcholine)	DN (CD4^−^CD8^−^) AC	MR Egger	19	0.095	1.216(0.979–1.510)	35.788
		Weighted median	19	0.085	1.126(0.984–1.289)	
		Inverse variance weighted	19	0.006	1.152(1.041–1.276)	
		Simple mode	19	0.070	1.270(0.996–1.619)	
		Weighted mode	19	0.055	1.149(1.006–1.313)	
DN (CD4^−^CD8^−^) AC	Rheumatoid Arthritis	MR Egger	16	0.073	1.156(0.999–1.339)	24.932
		Weighted median	16	0.064	1.081(0.995–1.173)	
		Inverse variance weighted	16	0.001	1.105(1.041–1.173)	
		Simple mode	16	0.926	1.007(0.863–1.177)	
		Weighted mode	16	0.702	1.029(0.892–1.186)

### 3.2 Association of liposomes with immune cells

**Fig. 3:**
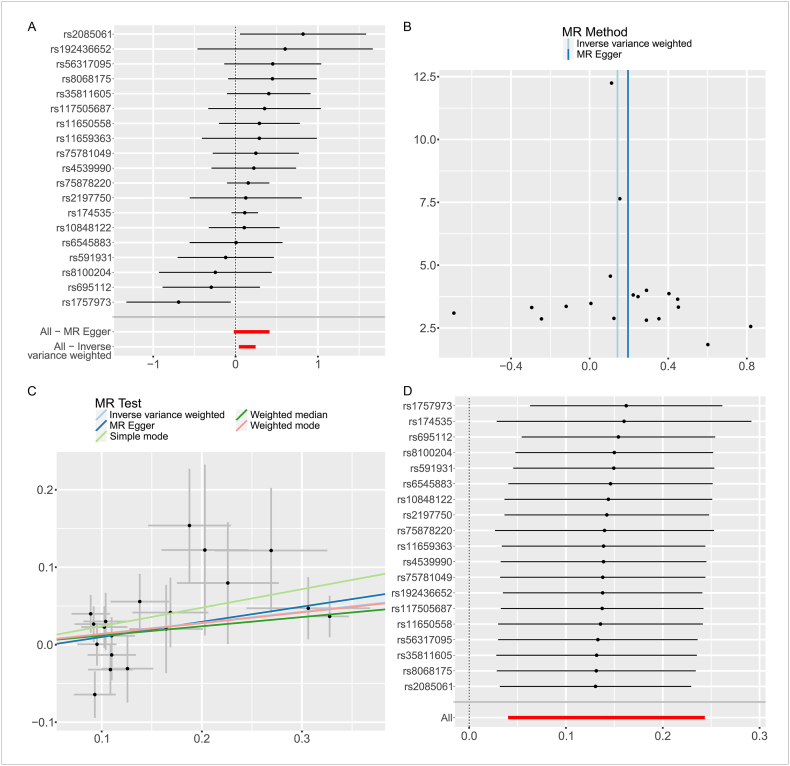
MR Analysis of phosphatidylcholine and DNAC. A: Leave-one-out forest map; B funnel diagram; C Scatter plot: Lines in black, red, green, and blue represent IVW, MR-Egger, weighted median, and weight mode methods; D Forest map for sensitivity analysis. (For interpretation of the references to colour in this figure legend, the reader is referred to the Web version of this article.)

The GWAS data of liposomes and immune cells showed a correlation between phosphatidylcholine and 731 immune cells (P < 0.05). Among them, DNAC (Double Negative CD4^−^CD8^−^ Autoreactive Cells) and phosphatidylcholine were the most strongly correlated, and 19 significantly correlated and independent SNPs were extracted for causal analysis. As shown in Table 1, the IVW method showed that increased levels of phosphatidylcholine were notably linked to higher levels of DNAC (P = 0.006, OR = 1.152, 95%CI = 1.041–1.276). Additionally, although they fell short of statistical significance, the results of the MR-Egger, Weighted median, Weighted mode, and Simple mode approaches indicated the same direction of causal estimate (MR-Egger: P = 0.095, OR = 1.216, 95%CI = 0.979–1.510; Weighted median: P = 0.085, OR = 1.126, 95%CI = 0.984–1.289; Simple mode: P = 0.070, OR = 1.270, 95%CI = 0.996–1.619; Weighted mode:P = 0.055, OR = 1.149, 95%CI = 1.006–1.313)(Fig. 3A–D).

### 3.3 Association of immune cells with RA

**Fig. 4:**
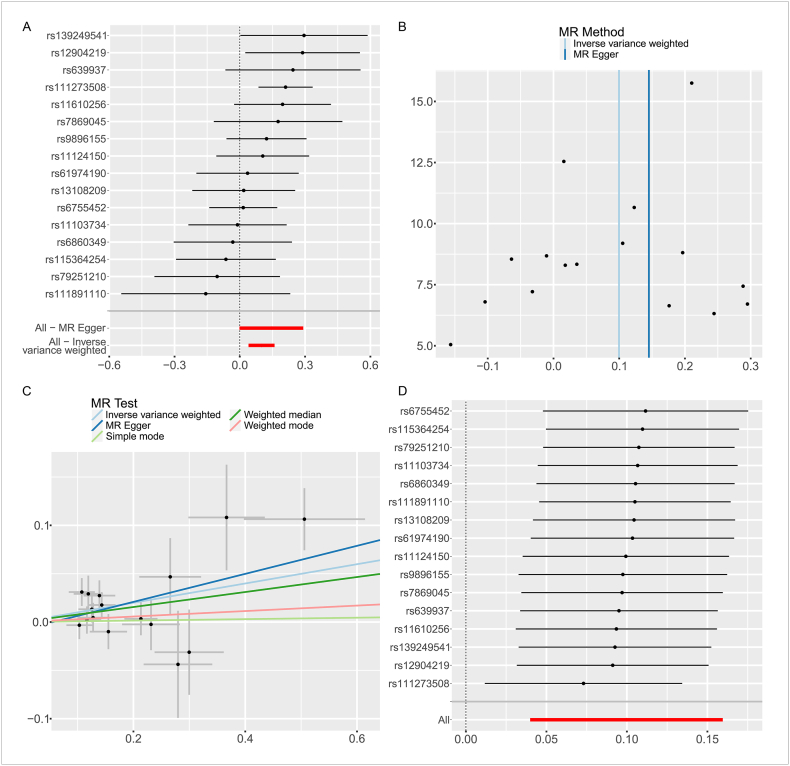
Results of MR Analysis of DNAC and RA. A: Leave-one-out forest map; B funnel diagram; C Scatter plot: Lines in black, red, green, and blue represent IVW, MR-Egger, weighted median, and weight mode methods; D Forest map for sensitivity analysis. (For interpretation of the references to colour in this figure legend, the reader is referred to the Web version of this article.)

DNAC were also found to be associated with RA risk from the GWAS aggregate data of immune cells, and 16 significant correlated and independent SNPs were extracted for causal analysis. As shown in Table 1, the IVW method showed that increased levels of DNAC were substantially linked to a higher risk of RA (P = 0.001, OR = 1.105, 95%CI = 1.041–1.173). The direction of causal estimation is consistent across the outcomes of the MR-Egger, Weighted median, Simple mode, and Weighted mode methods, but fail to reach statistical significance (MR-Egger:P = 0.072, OR = 1.156, 95%CI = 0.999–1.339; Weighted median: P = 0.064, OR = 1.081,95%CI = 0.995–1.173; Simple mode: P = 0.926, OR = 1.007, 95%CI = 0.863–1.177; Weighted mode: P = 0.702, OR = 1.029, 95%CI = 0.892–1.186)(Fig. 4A–D).

### 3.4 The association between immune cell mediated liposome and RA

**Fig. 5:**
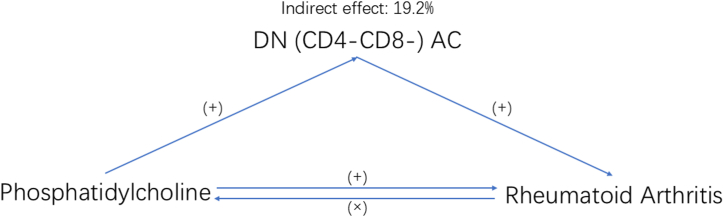
Schematic diagram of the DNAC mediation effect.

We found that an increase in liposomes was linked to a higher incidence of RA. At the same time, the rise in liposomes elevated DNAC levels, which ultimately led to an increased risk of RA. Our study suggests that the DNAC play a mediating role between liposome elevation and increased risk of RA. The intermediary effect is 19.2 %. (Fig. 5).

### 3.5 Sensitivity analysis

Cochran's Q test was used to assess heterogeneity, and the MR Egger intercept was applied to evaluate horizontal pleiotropy. The results, presented in Table 2, showed no signs of heterogeneity with all P-values exceeding 0.05. To visualize the heterogeneity of the studies, funnel plots were produced for each MR Analysis (Fig. 2, Fig. 3, Fig. 4, Table 2). There was no horizontal pleiotropy detected by the MR Egger intercept test (P > 0.05), and the MR-PRESSO global test did not find any outliers (Fig. 2, Fig. 3, Fig. 4, Table 2). Several sensitivity tests demonstrated the robustness of the MR results in this investigation (Fig. 2, Fig. 3, Fig. 4). The report also provides a forest plot showing each SNP's causal effect on outcome risk as IV (Fig. 2, Fig. 3, Fig. 4).

**Table 2:** The results of heterogeneity testing and pleiotropy testing.

Exposure	Outcome	Heterogeneity				Pleiotropy	
		MR Egger		IVW		MR Egger	
		*Q*	*P*	*Q*	*P*	Intercept	*P*
Phosphatidylcholine	Rheumatoid Arthritis	23.663	0.209	24.568	0.218	−0.008	0.405
Phosphatidylcholine	DN (CD4^−^CD8^−^) AC	18.992	0.329	19.333	0.372	−0.010	0.588
DN (CD4^−^CD8^−^) AC	Rheumatoid Arthritis	17.273	0.242	17.822	0.272	−0.008	0.516

### 3.6 Discussion

Recent research has examined the relationship between LP and RA. The available data, however, is confined to observational research and might be skewed by confounding variables. Our research clarified the causal link between RA and LP. This approach also helped determine whether their relationship is mediated by immune cells. Genetic predispositions to increased LP levels appear to elevate RA risk. Importantly, our analysis suggests that approximately 19.2 % of this effect is mediated through DNAC. This finding underscores the significance of immune modulation as a pathway, potentially opening new avenues for therapeutic intervention.

Our research indicates a positive correlation between elevated liposome levels and an increased risk of RA. However, direct studies specifically examining this relationship are currently lacking. Fuchs et al. [28] analyzed the ratio of phosphatidylcholine to lysophosphatidylcholine in patients' blood and found that as RA medication effectively controls the disease, this ratio increases. Since lysophosphatidylcholine is a direct metabolic product of phosphatidylcholine and its reduction is significantly more pronounced than that of phosphatidylcholine, the results suggest that phosphatidylcholine levels decrease correspondingly with the control of RA. This finding supports our research results, affirming a positive correlation between liposome and RA.

A well-known chronic autoimmune inflammatory disease, rheumatoid arthritis occurs when the immune system unintentionally targets and kills healthy joint tissue, leading to joint symptoms [3]. The pathogenesis of RA is closely linked to immune cell activity. Immune cells, including T cells and B cells, are inappropriately stimulated in individuals with RA, leading to the production of large quantities of autoantibodies. These autoantibodies, primarily rheumatoid factors of the IgM isotype, do not directly target joint tissues but bind to the Fc regions of IgG, forming immune complexes. These complexes can lodge in joints and other tissues, activating the complement system and triggering inflammation that results in tissue damage. Accompanied by inflammatory cytokines, this immune complex deposition promotes an inflammatory response in the synovial membrane, causing joint damage. DNAC, identified as CD4 CD8^−^ T cells, are a subtype of T cells. These cells typically express T cell receptors (TCRS) and can recognize their own antigens, triggering an immune response. They are rare in healthy individuals, constituting only 1%–2% of peripheral T lymphocytes, but they play a significant role in regulating immune responses. DNAC are crucial in autoimmune diseases, organ transplant immunity, and anti-tumor responses. Research by Liu et al. [29] found that DNAC levels in the synovial fluid of RA patients are notably higher than in their peripheral blood, suggesting a positive correlation between DNAC and RA. However, the specific mechanisms behind this phenomenon remain unexplored. This finding aligns with our research, which also demonstrates a positive correlation between DNAC and RA occurrence. Liposomes, as drug delivery systems, have shown significant potential in the treatment of RA by enhancing drug targeting, reducing systemic toxicity, and improving therapeutic efficacy. Wang et al. [30] discovered that polymeric stealth liposomes loaded with dexamethasone possess prolonged blood circulation times, effectively reducing inflammation in arthritic joints, thereby providing a promising drug delivery vehicle for various therapeutic applications. Chelvam et al. [31] found that follicular liposomes effectively targeted inflammation in the paws of arthritic rats, reducing paw swelling, arthritis scores, and bone erosion. Turker et al. [32]observed that treatment anti-inflammatory drugs for rheumatoid arthritis through intraarticular injection using drug delivery systems like liposomes/niosomes can prolong the residence time of the drugs in the joint.

This study has several limitations. First, the incidence of RA varies with ethnicity; to test our research, we limited the prevalence of RA to the European and Finnish populations. Second, a bigger dataset will be required for future research to confirm our findings because the number of cases in the RA genome-wide association studies (GWAS) sample is rather small. Third, we cannot totally rule out the chance that pleiotropy may have an impact on our results, even in spite of our best efforts to find and remove outlier variations. Fourth, our study utilized aggregate-level data rather than individual-level statistical data, limiting our ability to investigate causal linkages within subgroups, such as those observed between men and women. Fifth, our data indicates that IC is only responsible for 19.2 % of the genetic predisposition to rheumatoid arthritis, indicating the possibility of the involvement of other mediators. Finally, sensitivity analysis has some limitations in resolving pleiotropy. The statistical power of MR-Egger regression is low, especially when there are fewer instrumental variables. In addition, MR-Egger assumes that the pleiotropic effects of all instrumental variables are uniform, which may not always be true in practice. Cochran's Q test is sensitive to heterogeneity in the effects of instrumental variables, but cannot distinguish whether heterogeneity is due to pleiotropy or other factors (such as different sample sources).

## 4 Conclusion

We concluded that there is a causal association between liposomes and RA, with immune cells mediating 19.2 percent of the impact. However, the majority of the liposome influence on RA is yet unknown. More investigation is required into other risk variables that could serve as mediators.

## CRediT authorship contribution statement

**Huaguo Zhao:** Writing – review & editing, Writing – original draft, Software, Methodology, Data curation. **Licheng Ni:** Writing – review & editing, Visualization, Validation, Supervision, Project administration.

## Ethics approval and consent to participate

For this investigation, summary statistics from publicly available published studies and consortia were utilized. Consent was given by participants in the original research, and ethical approval was granted by relevant review boards. Given that this study did not involve individual data usage, no extra ethical approval was required.

## Consent for publication

Not applicable.

## Data availability statement

The research-related data has been stored in a publicly available repository.

## Exposure data

The exposure liposomes data in this study have been deposited in the GWAS catalog under accession codes Database ID ‘GCST90277238-GCST90277416’, comprising a total of 179 liposomes(URL: https://www.ebi.ac.uk/gwas/home).

## Immune cells data

The immune cells data in this study have been deposited in the GWAS catalog under accession codes Database ID ‘GCST90199621 to GCST90201020’, comprising a total of 731 immune cells(URL: https://www.ebi.ac.uk/gwas/home).

## Outcome data

The outcome data were obtained from the Finngen database and can be accessed at the following link: https://storage.googleapis.com/finngen-public-data-r9/summary_stats/finngen_R9_M13_RHEUMA.gz.

## Funding

No funding was received for this work from any governmental, private, or non-profit entities.

## Declaration of competing interest

The authors declare that they have no known competing financial interests or personal relationships that could have appeared to influence the work reported in this paper.
